# Modeling the Distribution of the Rare and Red-Listed Halophytic Moss Species *Entosthodon hungaricus* Under Various Climate Change Scenarios in Serbia

**DOI:** 10.3390/plants13233347

**Published:** 2024-11-28

**Authors:** Isyaku Abubakar, Jovana P. Pantović, Jasmina B. Šinžar-Sekulić, Marko S. Sabovljević

**Affiliations:** 1 Institute of Botany and Botanical Garden “Jevremovac”, Faculty of Biology, University of Belgrade, Takovska 43, 11000 Belgrade, Serbia; 2 Biotechnology Advanced Research Centre, Sheda Science and Technology Complex, P.M.B. 186, Garki, Abuja 900103, Nigeria; 3 Center of Plant Biotechnology and Conservation (CPBC), Takovska 43, 11000 Belgrade, Serbia; 4 Department of Plant Biology, Institute of Biology and Ecology, Faculty of Science, Pavol Jozef Šafárik University in Košice, Mánesova 23, 04001 Košice, Slovakia

**Keywords:** SDM (species distribution modeling), prediction, survival, conservation, threat, climate change

## Abstract

*Entosthodon hungaricus* is a rare moss species of the salty grasslands in Serbia. It is threatened with extinction due to habitat destruction and loss, although it reproduces sexually. In this study, we tested different models predicting its distribution under several climate scenarios over the next 8 decades. All models tested indicated a reduction in range to varying extents. Due to the specific substrate type as well as the predicted loss owing to the climate change, shifting is not an option for the survival of this species; and, therefore, it deserves special attention for its conservation and management.

## 1. Introduction

*Entosthodon hungaricus* (Boros) Loeske (syn. *E. maroccanum* (Meyl.) Hebr., *Funaria hungarica* Boros, *Physcomitrium maroccanum* Meyl, *P. martianovii* Broth.) was first described in Hungary in 1924 [1,2,3]. This xerophilic and photophilous species is considered an obligate halophyte, occurring in nature almost exclusively in saline, alkaline environments. However, laboratory experiments have shown that it can also develop on non-saline soils but in nature is not found in such situations due to its low competence with other non-halophytic bryophytes [4]. It usually grows on soils in halophytic steppes, in marginal areas of salt marshes, in saline areas near lagoons, and on the banks of alkaline lakes, but there are also some rare records from calcareous sandy areas and calcareous rocks in mountainous regions [5,6].

Analogous to other xerothermic species, this halophytic moss has a Continental-Mediterranean distribution [6,7]. Hodgetts and Lockhart [8] and Ellis et al. [9] reported the presence of this species in Austria, the Canary Islands, Germany, Greece, Hungary, Malta, Moldavia, Montenegro, Portugal, Romania, Russia, Serbia, Sicily, Slovakia, Spain, and Ukraine. Outside of Europe, it is also known in Israel, Kazakhstan, Kyrgyzstan, and Morocco [5]. However, despite its wide distribution, the range of *E. hungaricus* is very discontinuous, and the populations are highly scattered and fragmented [10,11].

As an ephemeral species, the phenological optimum of *E. hungaricus* is between April and May (also between February and July in exceptional cases), depending on the rainfall in early spring and the annual climatic fluctuations. The optimum observed in Serbia, for example, was between February and April [4]. It uses a short-lived life strategy to avert exceedingly unfavorable climatic conditions, e. g., cold and dry winters or dry and hot summers [12]. During this period, it actively produces sporophytes to quickly colonize suitable unoccupied habitats and leave the viable reproductive propagules in spore banks for the next good growing season.

Although it has a large distribution area throughout Europe, its range is very uneven. According to the new Red List of Mosses, Liverworts, and Hornworts, it is classified as Least Concern (LC) in Europe [13] mainly due to its high reproductive effort and almost always high spore production. However, no data are available on the spore viability, longevity, germination capacity, and/or dormancy of the spores. The European population as a whole is not considered to be threatened with extinction. However, due to its range, its short life span, its peculiar ecology, the specificity of the substrates, the habitat types, and the vulnerability of its habitats, it is on Red Lists in many European countries. It is classified as Critically Endangered in Slovakia (IUCN:CR), Highly Endangered in Austria, Vulnerable in Serbia (IUCN:VU), Near Threatened in Spain and Hungary (IUCN:NT), Data Deficient in the Canary Island and Malta (IUCN:DD), and Extremely Rare in Germany [11,14].

In Serbia, the known distribution is limited to saline grasslands in the province of Vojvodina (northern Serbia). Historically, this species was mentioned in the literature only by Guelmino [15,16,17,18], after which it was not recorded for nearly 40 years until new collections and localities were discovered by Papp et al. [19] and Sabovljević et al. [4].

Although it is relatively abundant in Serbian saline areas, it is a rare species in the country and threatened due to its ecology [19] and habitat degradation. In addition, the high vulnerability and sensitivity of saline habitats, which are affected by human activities and climate change in general, contribute to the species being threatened with extinction. These sites are particularly vulnerable due to their low agricultural value and are often used as low-class land for waste deposits and/or constructions, or sometimes as overgrazing pastures, which additionally affects rare species’ survival in such areas [4,19]. Severe climate change influences the specific water regime and, thus, the species survival, phenology, and development [4]. The sites of *E. hungaricus* in Serbia are habitat types of exceptional ecological and conservation importance. They are rare and endangered in Serbia and have a high conservation value not only for this species, but also as a habitat for some birds, insects, plants, and fungi of conservation interests [19]. Some of the risk factors observed in the field are overgrazing by cows in their growing sites and the degradation and/or destruction of their habitats [4]. According to the most recent Red List of Moss species of Serbia [14], the species is classified as VU (Vulnerable) based on recent distributions and species’ biology, while in the previous Red List of bryophytes in Serbia, it was classified as EN (Endangered) under criterion B [20]. However, in vitro cultivation, biological laboratory tests, ex situ collections, field research, and the partially successful strengthening of the population at a site in Serbia [4], as well as its proximity to a fairly large and good population in Hungary, have led to a recent decline in the conservation status of the species in Serbia.

Species distribution models (SDMs), which predict the distribution of species by modeling their ecological niche requirements [21,22], are used in many fields, including population evolution, global change ecology, and environmental conservation [23,24,25]. SDM uses algorithms to link the data of a species’ identified distribution points with environmental variables, and to create a model that estimates ecological requirements and predicts future distribution in a given area [26,27]. Various SDMs, including BIOCLIM, ENFA, CART, MaxEnt, GAM, RF, and GLM, are widely used in conservation biology, biogeography, and ecology [28,29,30].

Although the biology and ecology of *E. hungaricus* in Serbia have been studied to a certain extent, its distribution in the country is still not fully known. Considering that finding this species in the field can be challenging and time-consuming due to its small size and seasonality, SDMs represent a useful tool for discovering potential distribution areas of the species, especially in previously unexplored parts of the country. In addition, the prediction models can provide insight into the conservation management and monitoring of this species.

We used all known distribution data and climate data related to the recent distribution of this species in the country, as well as the available biological characteristics of this species (naturally obligate halophyte attached just to salt grasslands, large amount of spore production and, thus, huge spread potential in open flat areas to long distances), to create an SDM with the aim of (1) identifying suitable locations for its potential current distribution in the country, (2) predicting its future distribution range under different climate change scenarios, and (3) recognizing the main environmental variables affecting the distribution of this species in the country. The data generated through inferences on existing data owing to the use of SDMs will help us to better manage and conserve this species as well as its habitats.

## 2. Results

All algorithms used in this study effectively predicted the presence of *E. hungaricus* in Northern Serbia. The absence of the suitable substrates led to the exclusion of other parts of Serbia from further analyses. The mean ROC and TSS values across single-algorithm models were 0.981 ± 0.041 and 0.958 ± 0.080, respectively. Among the models, Random Forest (RF) achieved the highest ROC and TSS values, followed by Generalized Boosted Model (GBM), Generalized Linear Model (GLM), MAXNET, and MAXENT.

In the ensemble model created with the single-algorithm models that had a TSS above 0.7, 9 GLM, 11 GBM, 9 MAXENT, 12 MAXNET, and 9 RF models were included. The ROC and TSS of the ensemble in terms of committee averaging model were 0.997 and 0.99, respectively, and showed a better prediction ability compared to the single-algorithm models.

**Figure 1:**
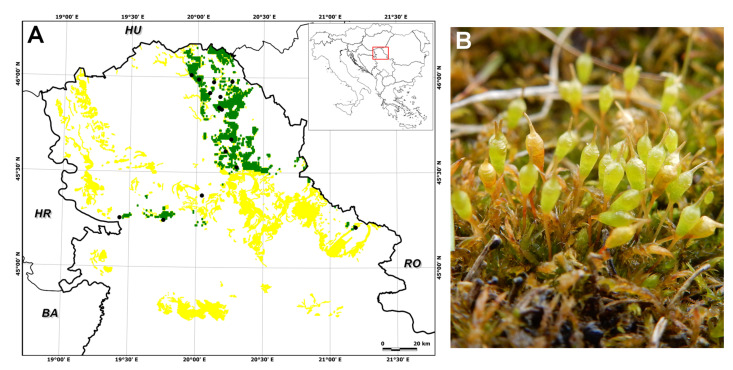
(**A**) The Vojvodina province (North of Serbia) within SE Europe. The yellow areas present the suitable substrates for *Entosthodon hungaricus* in Serbia, while green areas (overlapping with yellow) refer to those with optimal conditions for the present distribution of this species. Black dots indicate recent reports used for Species Distribution Modeling (BA—Bosnia and Herzegovina, HU—Hungary, HR—Croatia, RO—Romania). (**B**) Appearance of *E. hungaricus* in its natural habitat.

The suitable habitat area for *E. hungaricus* predicted by the ensemble model under the current climate conditions (1981–2010) was 1285 km^2^, and the habitat suitability was slightly higher compared to the currently known data on the distribution of this species (Figure 1). Considering that *E. hungaricus* is an obligate halophyte, halomorphic soil type (HAL) could not significantly influence distribution models in the time frame. Some of the climate conditions above the present salty areas under various climate scenarios and altitude did not have any influence. The greatest influence (of the eight predictor variables) on its distribution was the precipitation of the driest month (bio14) with a mean relative importance of 0.024 ± 0.007, followed by the altitude (ALT) with 0.003 ± 0.000, the mean daily air temperature range (bio2) with 0.003 ± 0.100, the precipitation seasonality (bio15) with 0.002 ± 0.00, the mean daily air temperatures of the warmest quarter (bio10) with 0.001 ± 0.000, the precipitation amount of the wettest month (bio13) with 0.001 ± 0.000, and the isothermality (bio3) with 0.001 ± 0.000.

**Figure 2:**
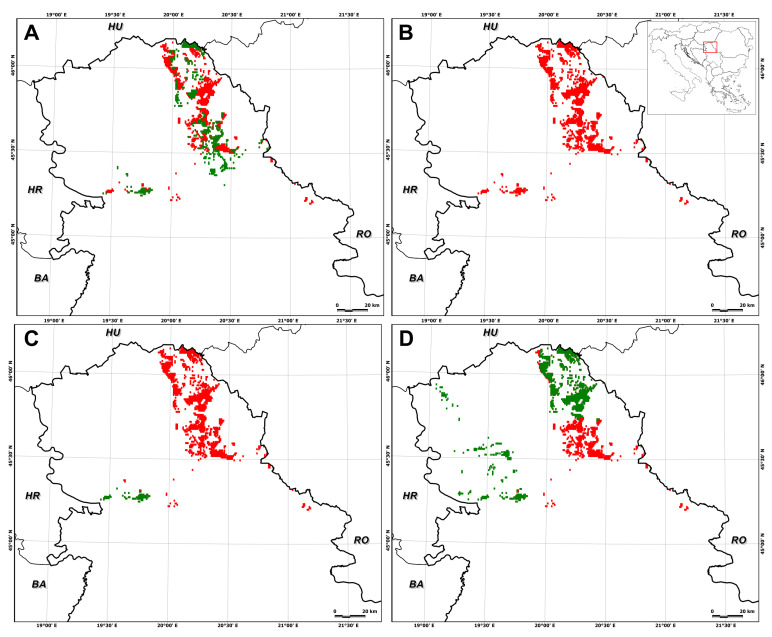
Mid-term distribution prediction (2041–2070) of *Entosthodon hungaricus* in Serbia, with a climate change scenario of a low-emission projection obtained for different global circulation models: (**A**) GFDL-ESM4; (**B**) UKESM1-0-LL; (**C**) MPI-ESM1-2HR; (**D**) IPSL-CM6A-LR). The green areas represent species predicted in the suitable distribution range, while red areas show a reduction in the suitable range.

**Figure 3:**
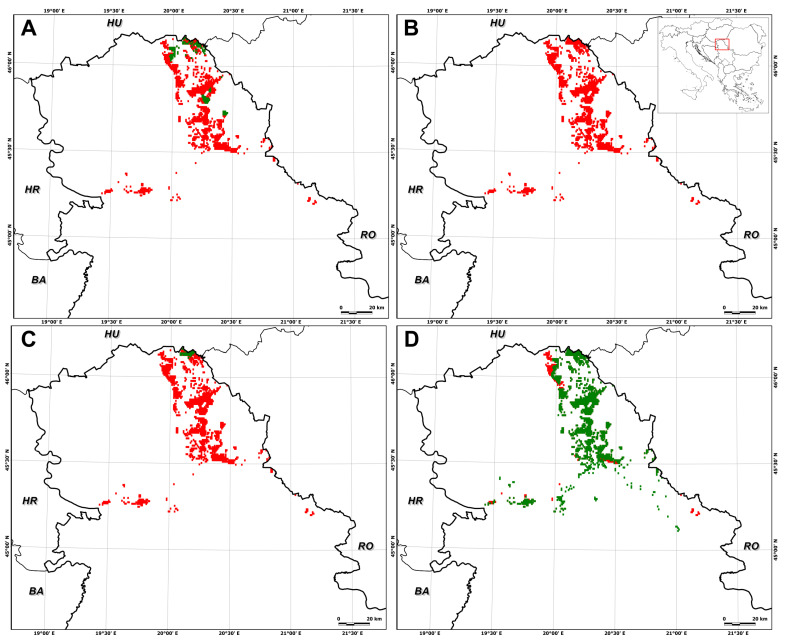
Long-term distribution prediction (2071–2100) of *Entosthodon hungaricus* in Serbia, with a climate change scenario of a low-emission projection obtained for different global circulation models: (**A**) GFDL-ESM4; (**B**) UKESM1-0-LL; (**C**) MPI-ESM1-2HR; (**D**) IPSL-CM6A-LR). The green areas represent species predicted in the suitable distribution range, while red areas show a reduction in the suitable range.

**Figure 4:**
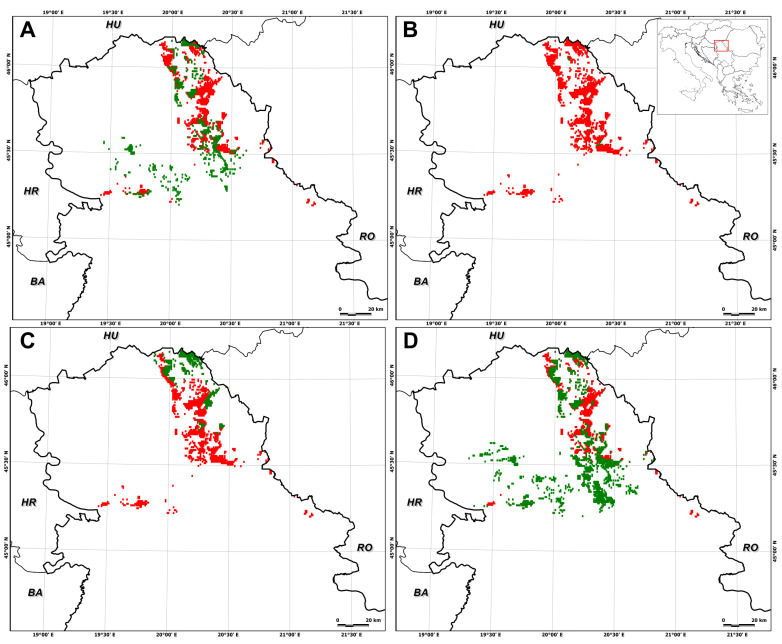
Mid-term prediction (2041–2070) of *Entosthodon hungaricus* in Serbia, with a climate change scenario of a high-emission projection obtained for different global circulation models: (**A**) GFDL-ESM4; (**B**) UKESM1-0-LL; (**C**) MPI-ESM1-2HR; (**D**) IPSL-CM6A-LR). The green areas represent species predicted in the suitable distribution range, while red areas show a reduction in the suitable range.

**Figure 5:**
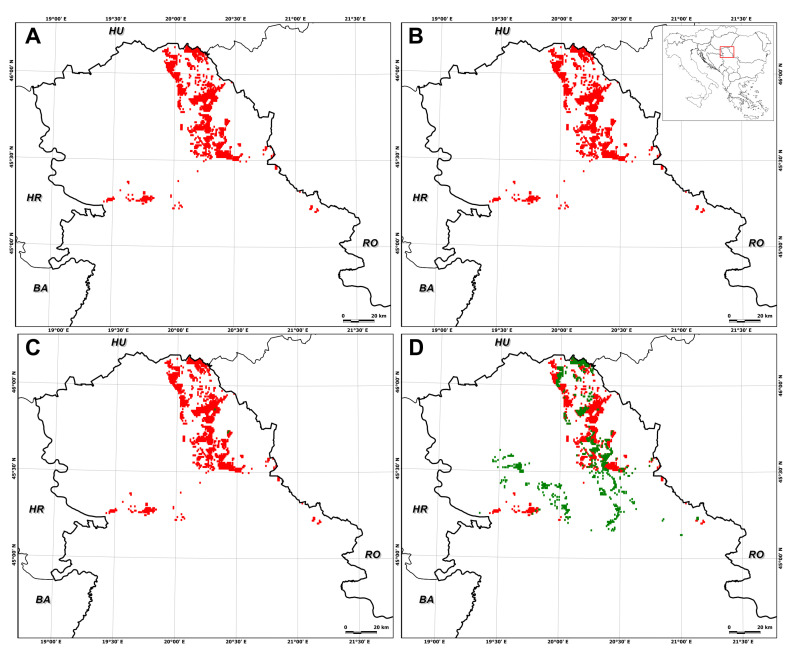
Long-term prediction (2071–2100) of *Entosthodon hungaricus* in Serbia, with a climate change scenario of a high-emission projection obtained for different global circulation models: (**A**) GFDL-ESM4; (**B**) UKESM1-0-LL; (**C**) MPI-ESM1-2HR; (**D**) IPSL-CM6A-LR). The green areas represent species predicted in the suitable distribution range, while red areas show a reduction in the suitable range.

In particular, suitable habitat for *E. hungaricus* is predicted to decrease by 75.0 ± 27.5% (SSP1–2.6, 2041–2070), 74.0 ± 42.7% (SSP1–2.6, 2071–2100), 72.9 ± 21.9% (SSP5–8.5, 2041–2070), and 92.0 ± 15.8% (SSP5–8.5, 2071–2100) (Figure 2, Figure 3, Figure 4 and Figure 5). Conversely, certain areas that are currently unsuitable for the species are expected to become suitable under future climate conditions, with a range expansion of 4.7 ± 5.6% (SSP1–2.6, 2041–2070), 2.5 ± 5.1% (SSP1–2.6, 2071–2100), 17.1 ± 23.8% (SSP5–8.5, 2041–2070), and 5.8 ± 11.7% (SSP5–8.5, 2071–2100) (Figure 2, Figure 3, Figure 4 and Figure 5).

Under the low-emission scenario, the prediction range reduced in mid-term models as inferred by the data under future climate scenarios, with average declines ranging from −70.3 ± 33.0% under SSP1–2.6 within 2041–2070 (Figure 2) to −71.52 ± 47.7% under SSP1–2.6 within 2071–2100 (Figure 3).

The analysis of range shifts under the high-emission scenario revealed that E. hungaricus is predicted to experience significant range reductions under future climate scenarios, with average declines ranging from −55.8 ± 44.4% under SSP5–8.5 in the period of 2041–2070 (Figure 4) to −86.28 ± 27.4% under SSP5–8.5 in the period of 2071–2100 (Figure 5). These shifts are likely to be primarily due to the loss of suitable habitats under future climate conditions.

As for the individual global circulation models (GCMs), the different GCMs provided different predictions for future habitat suitability in terms of decreases and increases in suitable areas. The largest decrease in suitable habitat area was observed for UKESM1-0-LL for both time periods and SSPs, while the largest increase, also for both time periods and SSPs, was observed for IPSL-CM6A-LR (Figure 1, Figure 2, Figure 3, Figure 4 and Figure 5).

## 3. Discussion

The worldwide rise in temperature is threatening the viability of global ecosystems, which is affecting biodiversity across regions [31,32]. Climate change is causing species to relocate to higher altitudes and latitudes [33,34,35], and as species migrate to higher altitudes, rare and endangered species with limited geographic ranges, tiny population densities, or high habitat selectivity are at risk of extinction as their ranges decline [36,37,38].

Indeed, we show here that all prediction models lead to a significant reduction in the Serbian population of *Entosthodon hungaricus* within the next 8 decades due to severe environmental changes. This is evidence that the survival of the species in Serbia will be at risk in the future and should also have an impact on its conservation plans and measures. Although there is a possibility that the population will shift from the currently drier Banat (east of Vojvodina) to the currently slightly wetter Bačka (west of Vojvodina), the probability is low, and the range will not increase, on the contrary, as the eastern areas will be too dry for the survival of this species.

Even though the IPSL-CM6A-LR model appears to be the most optimistic compared to the other three tested, it should be taken with caution. It has been shown that this model is not suitable for species with narrow ecological requirements, as was the case with *E. hungaricus*, as the spatial resolution is rather low [39]. Thus, the future and the survival and distribution of this species are not very promising.

According to the results obtained, the salt substrate is the crucial factor for this species’ survival. As considered in relation to climatic features, the precipitation of the driest month, precipitation seasonality, and mean diurnal temperature range seem to be among the most effective factors influencing the species’ development. Indeed, *E. hungaricus* in Serbia was documented during the wet late winter and early spring season, and the rather high temperature and dry spring deviations in some years leaded to decrease the short-lived species’ appearances and occurrences as previously documented [4]. The one-season deviation did not significantly affect the development next year after the deviating season due to a spore bank. The successional year’s spring climate deviation led to certain site species’ occurrences decreasing during the next adequate developmental season [4].

SDM models can be particularly significant for ephemeral species that can be easily overlooked in the field or that are difficult to identify [40]. This was the case for tested moss *E. hungaricus*, and thus, we confirm the suitability of such models for narrow niche ephemeral moss species as well. These models, therefore, help us to determine suitable habitats and the potential current distribution in the area of interest. The test results will surely be confirmed through a visit and the species’ documentation within the next suitable period. To date, there have been no studies investigating the factors influencing the development and (potential) distribution of *E. hungaricus* in its natural environment. Such studies are also important because they allow for predictions to be made about the future distribution of the species, which is crucial for its conservation. The already documented trend of temperature increase in the territory of Serbia in combination with a decrease in precipitation and/or uneven annual distribution [41] confirms that the scenario of loss of the majority of current habitats for *E. hungaricus* in Serbia in the future is quite realistic.

The conservation status of this species in Serbia is considered as vulnerable (VU) [14]. Due to the high reproductive effort and spore production often present in this species, its extinction risk in Europe decreased from vulnerable (VU) to least concern (LC) in the latest threat assessment [11,13]. Though, in previous assessments, the climate change effects and anthropogenic salty grassland degradation (as lands of low economic benefits) were underestimated. These are, however, rather important issues for species survival as shown by our results.

## 4. Materials and Methods

### 4.1. Study Area and Species Occurrences

The region selected for this study was the Republic of Serbia, a country in the central part of the Balkan Peninsula in the south-eastern part of Europe. It is characterized by a heterogenous terrain and a complicated relief, with a very complex and diversified climate due to Pannonian, Mediterranean, and Atlantic influences [42]. As a result, the climate varies between a dry continental climate in the north, a humid version in the west, a mild continental climate in the central part, and a very dry sub-Mediterranean climate in the south-southeastern part of the country. In addition, the mountainous parts of the country are characterized by a distinct mountain climate. Influenced by the prevailing weather trends, the total amount of precipitation varies and can range from approx. 500 mm in Northern Vojvodina to approx. 1000 mm in Western Serbia and over 1500 mm in high-mountain regions.

The province of Vojvodina occupies the northernmost part of the country and is separated from the central part of Serbia by the Sava and Danube rivers. It is located in the southern part of the Pannonian Plain. It consists of three regions: Banat, Bačka, and Srem. Most of Vojvodina is lowland with an altitude between 68 and 120 m, although there are two distinct mountain formations, namely Vršačke Planine (639 m) in Banat and Fruška Gora (538 m) in Srem [43]. The hydrographic system consists of large rivers such as the Danube and its tributaries Sava and Tisa (Tisza), which have had a significant influence on the geomorphologic development of Vojvodina. The province of Vojvodina is characterized by a continental climate distinguished by large temperature extremes, cold winters, and the presence of arid and semi-arid periods during the summer and early autumn months. The average annual precipitation is 520–590 mm [44,45]. Vojvodina belongs entirely to the Pannonian biogeographical region and is a secondary forest-steppe area. However, forests and wooded areas cover 6.8% of the territory [46].

According to Vidojević et al. [47], some of the typical soil types in Serbia are Vertisols, Gleysol, Chernozems, Calcisol, Cambisol, Fluvisol, and Solonchak soils. Chernozems, Gleysol, and Solonchaks are the most common soil reference groups in the province of Vojvodina. The most saline soils are solonchaks, which contain at least 1% salt, with sodium carbonate (Na_2_CO_3_) being the main salt type in the solonchaks of Vojvodina, while sulfates (mainly Na_2_SO_4_) and chlorides (mainly NaCl) are present to a lesser extent [48].

The records of *E. hungaricus* in Serbia were compiled from various sources in the literature [4,15,16,17,18,19] and the bryophyte collection of the Herbarium of the University of Belgrade (BEOU). Most of the old literature records were confirmed as recent prior to application in this study. A total of 22 occurrences of the species in Serbia were documented. In order to reduce sampling bias, a suitable setting was made during data preparation using the R package biomod2, version 4.2-4 [49], to obtain only one occurrence point per environmental grid cell. After the bias reduction process, 16 records were retained for the final modeling. Although the number of occurrences seems to be small, it is rather valuable and useful and has been previously elaborated in other case studies [50]. It is considered that this size is sufficient for most algorithms to perform satisfactorily during the modeling process. Additionally, *E. hungaricus* is a specialist, i.e., an obligate halophyte, that lives in saline, alkaline environments in nature and, thus, is a species with a narrow ecological niche.

### 4.2. Environmental Variables

The climate data set comprises 19 bioclimatic variables from CHELSA (Climatologies at High Resolution for Earth’s Land Surface Areas) version 2.1, with a spatial resolution of 30 arc seconds (~1 km) [51,52]. Four global circulation models (GCMs)—GFDL-ESM4, UKESM1-0-LL, MPI-ESM1-2HR, and IPSL-CM6A-LR—from the CMIP6 (Coupled Model Intercomparison Project Phase 6) were selected based on their compliance with the ISIMIP3b protocol [52]. Three time periods were analyzed: 1981–2010 for the present climate and 2041–2070 and 2071–2100 for future projections. In addition, two socio-economic pathways (SSPs)—SSP1–2.6 (low-emission projections, ca. +1.8 °C by 2100) and SSP5–8.5 (high-emission projections, ca. +4.4 °C by 2100) [53]—were used to predict the potential impact of climate change on *E. hungaricus* [54,55].

In addition to the bioclimatic variables, two non-climatic variables were included: altitude, derived from SRTM elevation data [56], and a soil type variable representing halomorphic soils, derived from a binary grid where values of 1 indicate the presence and 0 the absence of halomorphic soils [57]. Both variables have a spatial resolution of 30 arc seconds (~1 km).

To avoid multicollinearity, Pearson’s correlation coefficients were calculated for all environmental variables at the sites where *E. hungaricus* was observed in Serbia using the usdm package in R [58]. Variables with correlation coefficients (r) greater than 0.7 were excluded from further analysis. Retained variables included mean daily temperature range (bio2), isothermality (bio3), mean daily air temperatures of the warmest quarter (bio10), precipitation of the wettest month (bio13), precipitation of the driest month (bio14), precipitation seasonality (bio15), altitude (ALT), and halomorphic soils (HAL).

### 4.3. Modeling the Species Distribution

Ensemble modeling was used to assess the current distribution of *E. hungaricus* and predict future distribution shifts under the influence of climate change. This approach was chosen because ensemble modeling has been shown to increase predictive accuracy and reduce overfitting, especially in models for rare and endangered species [59].

Ensemble modeling was performed using the biomod2 version 4.2-4 package in R [49]. Five algorithms were used: Generalized Linear Model (GLM); Generalized Boosted Model, i.e., boosted regression trees (GBM); Maximum Entropy (MAXENT and MAXNET); and Random Forest (RF). For each algorithm, the models were run on three pseudo-absence datasets, each with 160 randomly selected points (ten times the number of occurrence points) with five iterations per dataset. Cross-validation was performed by randomly splitting the data into training (80%) and testing (20%) datasets and was repeated ten times to ensure robust evaluation of model performance.

Model performance was assessed using the True Skill Statistic (TSS) [60] and the Area Under the Receiver Operating Characteristic Curve (ROC) [61]. Only models with TSS values above 0.7 were included in the final ensemble modeling. Committee Averaging (EMca) was selected as the ensemble modeling strategy due to its superior predictive performance, as evidenced by its highest ROC and TSS values obtained [62].

For the binary classification of suitable and unsuitable habitat, a TSS-based threshold was used, which was determined by evaluating different thresholds and selecting the one that maximizes the TSS [49]. The biomod2 package was also used to assess the changes in species’ range under different climate scenarios. Initial and generated data sets are available upon request.

## 5. Conclusions

Understanding how rare and endangered species respond to climate change is crucial for assessing threats to biodiversity and planning conservation measures. The adequate substrates under climate changes are the main factors affecting the moss *E. hungaricus* survival and distributions. As the species is rare and threatened in Serbia, the tested models enable the search for new suitable microhabitats and subpopulations in the country. The habitat types, mainly salty grasslands with no high vegetation biomass production, should be better maintained and managed. This is rather an easier task compared to coping with climate changes, where suitable microhabitats should be selected for further ex situ conservation, and new population establishments. However, all tested species’ distribution models for the rare halophytic moss *Entosthodon hungaricus* in Serbia showed negative trends, i.e., population and subpopulation decrease. This means that special attention needs to be paid to the survival of the species, as well as to establishing monitoring and conservation plans, to prevent the regional species’ loss.

## Data Availability

Data are contained within the article.

## References

[1] Boros A. (1924). *Funaria hungarica*, nov. spec. Magy. Bot. Lapok.

[2] Cano M.J., Ros R.M., Guerra J., Conzilez J. (1999). The identity of *Entosthodon hungaricus* (Boros) Loeske and *E. maroccanus* (Meyl.) Hebr. & Lo Giudice (=*Physcomitrium maroccanum* Meyl.). J. Bryol..

[3] Ignatova E.A., Ignatov M.S. (2005). On the identity of *Physcomitrium martianovii* (Funariaceae, Bryophyta). Arctoa.

[4] Sabovljević M.S., Nikolić N., Vujičić M., Šinžar-Sekulić J., Pantović J., Papp B., Sabovljević A.D. (2018). Ecology, distribution, in vitro propagation, ex situ conservation and native population strenghtening of the rare and threatened halophyte moss *Entosthodon hungaricus* in Serbia. Wulfenia.

[5] Pisarenko O.Y., Ignatova E.A., Ignatov M.S. (2001). *Entosthodon hungaricus* (Boros) Loeske (Funariaceae, Musci) in Altaisky territory, South Siberia. Arctoa.

[6] Papp B. (2002). New records of bryophytes from a saline area of Greece. Stud. Bot. Hung.

[7] Pocs T., Sabovljevic M., Puche F., Segarra-Moragues J.G., Gimeno C., Kürschner H. (2004). *Crossidium laxefilamentosum* Frey and Kurschner (Pottiaceae), new to Europe and to North Africa. Studies on the cryptogamic vegetation on loess clifs, VII. J. Bryol..

[8] Hodgetts N., Lockhart N. (2020). Checklist and Country Status of European Bryophytes–Update 2020.

[9] Ellis L.T., Aleffi M., Alegro A., Šegota V., Asthana A.K., Gupta R., Singh V.J., Bakalin V.A., Bednarek-Ochyra H., Cykowska-Marzencka B. (2016). New national and regional bryophyte records, 48. J. Bryol..

[10] Sabovljevic M., Papp B., Sabovljevic A., Vujicic M., Szurdoki E., Segarra-Moragues J.G. (2012). In vitro micropropagation of rare and endangered moss *Entosthodon hungaricus* (Funariaceae). Biosci. J..

[11] Sabovljevic M., Papp B., Blockeel T., Ignatov M., Hallingbäck T., Söderström L., Entosthodon Hungaricus (Europe Assessment) The IUCN Red List of Threatened Species 2019: e.T85841487A87779152. https://www.iucnredlist.org/species/85841487/87779152.

[12] Hebrard J.P., Giudice R.L. (1996). *Physcomitrium maroccanum* Meylan in Sicily, new to European bryoflora. J. Bryol..

[13] Hodgetts N., Cálix M., Englefield E., Fettes N., Criado M.G., Patin L., Nieto A., Bergamini A., Bisang I., Baisheva E. (2019). Miniature World in Decline: European Red List of Mosses, Liverworts and Hornworts.

[14] Sabovljević M.S., Pantović P.J., Širka P., Vujičić M.M., Sabovljević D.A., Papp B. (2024). Red-list of moss species of Serbia: 2024 assessment. Bot. Serb..

[15] Guelmino J. (1970). Prva nalazišta mahovine *Funaria hungarica* Boros u Jugoslaviji. Zb. Za Prir. Nauk. Matica Srp..

[16] Guelmino J. (1972). Nova nalazišta dve malo poznate mahovine kod nas (*Funaria hungarica* Boros i *Tortula velenovsky* Schiffner). Zb. Za Prir. Nauk. Matica Srp..

[17] Guelmino J. (1973). Zenta es kornyekenek novenyei II. (Viragtalanok). [Plants of Senta and its surrounding II. (Cryptogams)]. Građa Za Monogr. Sente.

[18] Guelmino J. (1997). Ritka zuzmók és mohák nyomában vajdaság területén. Híd Irod. Művészeti És Társadalomtudományi Folyóirat.

[19] Papp B., Alegro A., Erzberger P., Szurdoki E., Šegota V. (2016). Bryophytes of saline areas in the Pannonian region of Serbia and Croatia. Stud. Bot. Hung..

[20] Sabovljevic M., Cvetic T., Stevanovic V. (2004). Bryophyte red list of Serbia and Montenegro. Biodivers. Conserv..

[21] Pulliam H.R. (2000). On the relationship between niche and distribution. Ecol. Lett..

[22] Peterson A.T., Soberón J. (2012). Integrating fundamental concepts of ecology, biogeography, and sampling into effective ecological niche modeling and species distribution modeling. Plant Biosyst..

[23] Chen X.D., Yang J., Feng L., Zhou T., Zhang H., Li H.M., Bai G.Q., Meng X., Li Z.H., Zhao G.F. (2020). Phylogeography and population dynamics of an endemic oak (*Quercus fabri* Hance) in subtropical China revealed by molecular data and ecological niche modeling. Tree Genet. Genomes.

[24] Guisan A., Zimmermann N.E. (2000). Predictive habitat distribution models in ecology. Ecol. Model..

[25] Peterson A.T., Papes M., Eaton M. (2007). Transferability and model evaluation in ecological niche modeling: A comparison of GARP and Maxent. Ecography.

[26] Kulhanek S.A., Leung B., Ricciadi A. (2011). Using ecological niche models to predict the abundance and impact of invasive species: Application to the common carp. Ecol. Appl..

[27] Dyderski M.K., Paz S., Frelich L.E., Jagodzinski A.M. (2018). How much does climate change threaten European forest tree species distributions?. Glob. Change Biol..

[28] Gaston K.J. (1996). Species-range-size distributions: Patterns, mechanisms and implication. Trends Ecol. Evol..

[29] Kenar N., Kikvidze Z. (2023). Modelling the distribution of the Caucasian oak (*Quercus macranthera*) in Western Asia under future climate change scenarios. Bot. Serb..

[30] Bîrsan C., Mardari C., Copoţ O., Tănase C. (2021). Modelling the potential distribution and habitat suitability of the alien fungus Clathrus archeri in Romania. Bot. Serb..

[31] Dawson T.P., Jackson S.T., House J.I., Prentice I.C., Mace G.M. (2011). Beyond predictions: Biodiversity conservation in a changing climate. Science.

[32] Purves D.W., Dushoff J. (2005). Directed seed dispersal and metapopulation response to habitat loss and disturbance: Application to *Eichhornia paniculata*. J. Ecol..

[33] Chen I.C., Hill J.K., Ohlemuller R., Roy D.B., Thomas C.D. (2011). Rapid range shifts of species associated with high levels of climate warming. Science.

[34] He X., Burgess K.S., Yang X.F., Ahrends A., Gao L.M., Li D.Z. (2019). Upward elevation and northwest range shifts for alpine Meconopsis species in the Himalaya-Hengduan Mountains region. Ecol. Evol..

[35] Parmesan C., Yohe G. (2003). A globally coherent fingerprint of climate change impacts across natural systems. Nature.

[36] Jump A.S., Peuelas J. (2005). Running to stand still: Adaptation and the response of plants to rapid climate change. Ecol. Lett..

[37] Pauli H., Gottfried M., Dullinger S., Abdaladze O., Grabherr G. (2012). Recent plant diversity changes on Europe’s Mountain summits. Science.

[38] Daskalova G.N., Myers-Smith I.H., Godlee J.L. (2020). Rare and common vertebrates span a wide spectrum of population trends. Nat. Commun..

[39] Boucher O., Servonnat J., Albright A.L., Aumont O., Balkanski Y., Bastrikov V., Bekki S., Bonnet R., Bony S., Bopp L. (2020). Presentation and evaluation of the IPSL-CM6A-LR climate model. J. Adv. Model. Earth Syst..

[40] Sérgio C., Figueira R., Draper D., Menezes R., Sousa A.J. (2007). Modelling bryophyte distribution based on ecological information for extent of occurrence assessment. Biol. Conserv..

[41] Dimkić D. (2019). Characteristics of annual, seasonal and monthly observed climate and hydrologic changes in Serbia in the last seventy years. Desalin. Water Treat..

[42] Lalić B., Eitzinger J., Mihailović D.T., Thaler S., Jančić M. (2013). Climate change impacts on winter wheat yield change–which climatic parameters are crucial in Pannonian lowland?. J. Agric. Sci..

[43] Živković B., Nejgebauer V., Tanasijević Đ., Miljković N., Stojkoivć L., Drezgić P. (1972). Soils of Vojvodina.

[44] Stevanović V., Stevanović B., Stevanović V., Vasić V. (1995). Osnovni klimatski, geološki i pedološki činioci biodiverziteta kopnenih ekosistema Jugoslavije. Biodiverzitet Jugoslavije: Sa Pregledom Vrsta od Međunarodnog Značaja.

[45] Smailagić J., Savović A., Marković D., Nešić D., Drakula B., Milenković M., Zdravković S. (2013). Climate Characteristics of Serbia.

[46] Trišić I., Štetić S., Maksin M. (2020). The significance of protected natural areas for tourism in the Vojvodina Province (Northern Serbia)—Analysis of sustainable tourism development. Spatium.

[47] Vidojević D., Manojlović M., Đorđević A., Nešić L., Dimić B. (2016). Organic Carbon Stocks in the Chernozems of Serbia.

[48] Miljković N. (2005). Meliorativna pedologija.

[49] Thuiller W., Georges D., Gueguen M., Engler R., Breiner F., Lafourcade B., Patin R. (2023). biomod2: Ensemble Platform for Species Distribution Modeling. https://CRAN.R-project.org/package=biomod2.

[50] Sillero N., Arenas-Castro S., Enriquez-Urzelai U., Vale C.G., Sousa-Guedes D., Martínez-Freiría F., Real L., Barbosa A.M. (2021). Want to model a species niche? A step-by-step guideline on correlative ecological niche modelling. Ecol. Modell..

[51] Brun P., Zimmermann N.E., Hari C., Pellissier L., Karger D.N. (2022). Global climate-related predictors at kilometer resolution for the past and future. Earth Sys. Sci. Data.

[52] Karger D.N., Conrad O., Böhner J., Kawohl T., Kreft H., Soria-Auza R.W., Zimmermann N.E., Linder H.P., Kessler M. (2017). Climatologies at high resolution for the earth’s land surface areas. Sci. Data.

[53] Blais B.R., Koprowski J.L. (2024). Modeling a hot, dry future: Substantial range reductions in suitable environment projected under climate change for a semiarid riparian predator guild. PLoS ONE.

[54] Meinshausen M., Nicholls R.Z., Lewis J., Gidden J.M., Vogel E., Freund M., Beyerle U., Gessner C., Nauels A., Bauer N. (2000). The shared socio-economic pathway (SSP) greenhouse gas concentrations and their extensions to 2500. Geosci. Model Dev..

[55] Tebaldi C., Debeire K., Eyring V., Fischer E., Fyfe J., Friedlingstein P., Knutti R., Lowe J., O’Neill B., Sanderson B. (2021). Climate model projections from the Scenario Model Intercomparison Project (ScenarioMIP) of CMIP6. Earth Syst. Dynam..

[56] WorldClim (2020). WorldClim 2.1: 30 Arc Seconds SRTM Elevation Data. https://www.worldclim.org/data/index.html.

[57] Pavlović P., Kostić N., Karadžić B., Mitrović M. (2017). The Soils of Serbia.

[58] Naimi B., Hamm N.A., Groen T.A., Skidmore A.K., Toxopeus A.G. (2014). Where is positional uncertainty a problem for species distribution modelling?. Ecography.

[59] Breiner F.T., Guisan A., Bergamini A., Nobis M.P. (2015). Overcoming limitations of modelling rare species by using ensembles of small models. Methods Ecol. Evol..

[60] Allouche O., Tsoar A., Kadmon R. (2006). Assessing the accuracy of species distribution models: Prevalence, kappa and the true skill statistic (TSS). J. Appl. Ecol..

[61] Jiménez-Valverde A. (2012). Insights into the area under the receiver operating characteristic curve (AUC) as a discrimination measure in species distribution modelling. Global Ecol. Biogeogr..

[62] Mohajane M., Costache R., Karimi F., Pham Q.B., Essahlaoui A., Nguyen H., Laneve G., Oudija F. (2021). Application of remote sensing and machine learning algorithms for forest fire mapping in a Mediterranean area. Ecol. Indic..

